# Supplementary material for: Solving groundwater depletion in India while achieving food security
Source: Nat Commun. 2022 Jun 13;13:3374. doi: 10.1038/s41467-022-31122-9 (PMC9192749; doi:10.1038/s41467-022-31122-9)
Supplement: Supplementary file 1 — Supplementary Information [file 41467_2022_31122_MOESM1_ESM.pdf]

Solving Groundwater Depletion in India while achieving Food Security

Supplementary Information

Naresh Devineni[1,2*], Shama Perveen[3] and Upmanu Lall[2,4]

[1]Department of Civil Engineering, City University of New York (City College), New York, NY 10031, USA

[2]Columbia Water Center, Columbia University, New York, NY 10027, USA

[3]CERES, 99 Chauncy St. 6th Floor, Boston, MA 02111 (research done during the affiliation with Columbia University)

[4]Department of Earth and Environmental Engineering, Columbia University, New York, NY 10027, USA

*Corresponding Author: Naresh Devineni e-mail: ndevineni@ccny.cuny.edu

Supplementary Note 1

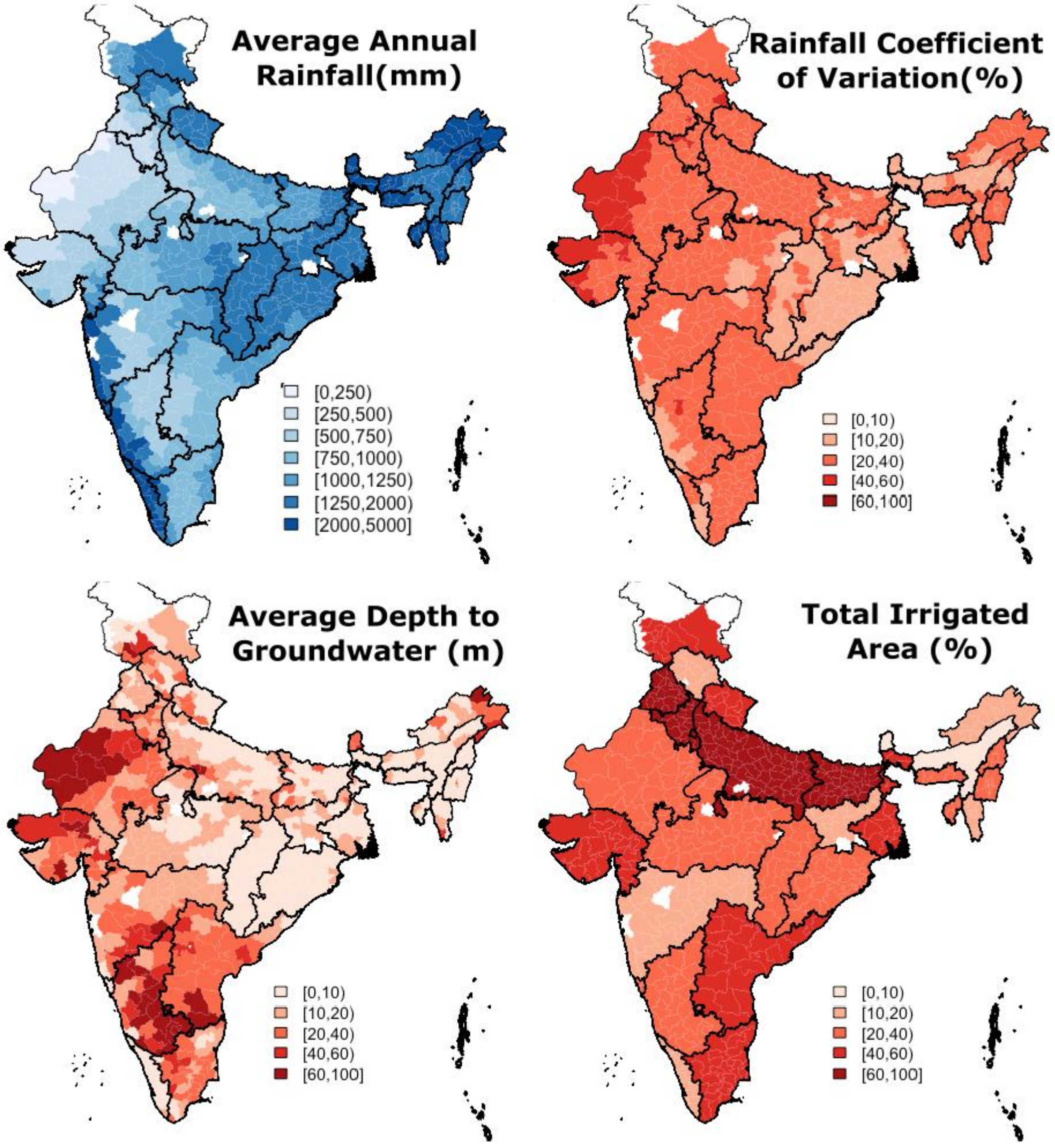

Fig. S1 The district-level average annual rainfall (top left panel), the inter-annual coefficient of variation of rainfall (top right panel), the average depth to groundwater level (bottom left panel), and the state-wide percentage total irrigation area coverage under all the crops (bottom right panel).

Supplementary Note 2

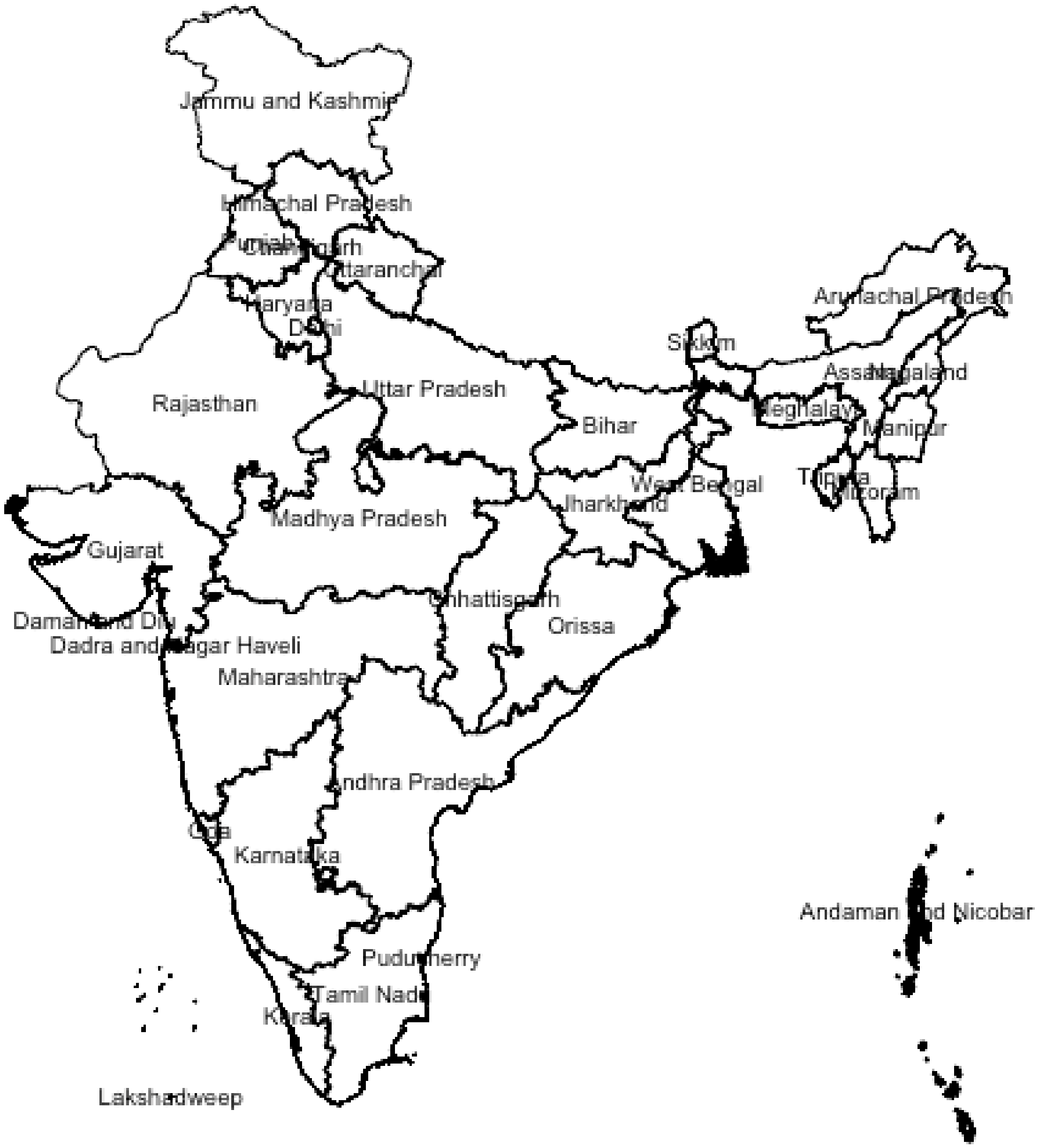

Fig. S2 A map showing Indian states based on ArcMap's 2001 state boundary shapefile.

Supplementary Note 3

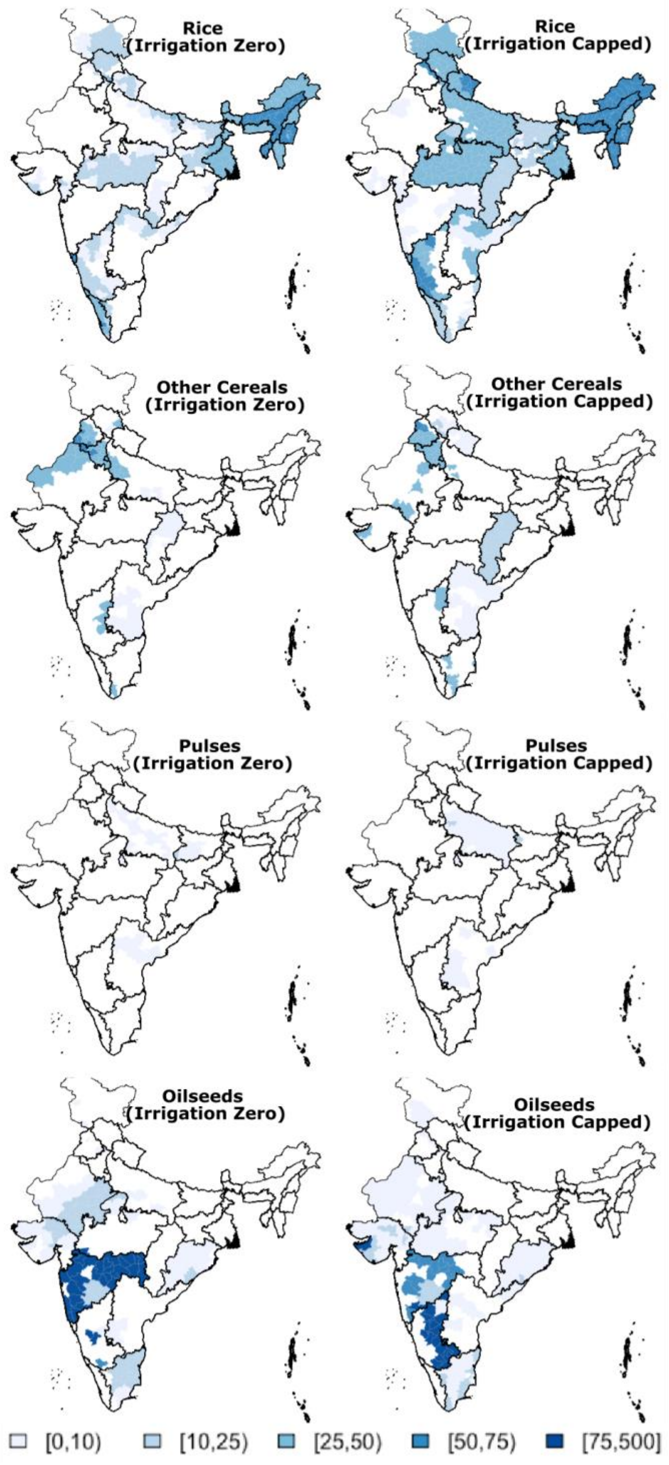

Fig. S3 Maximum allowable percent decrease in the minimum support price of rice, other cereals, pulses and oilseeds while maintaining the proposed optimal cropping patterns.

Supplementary Note 4

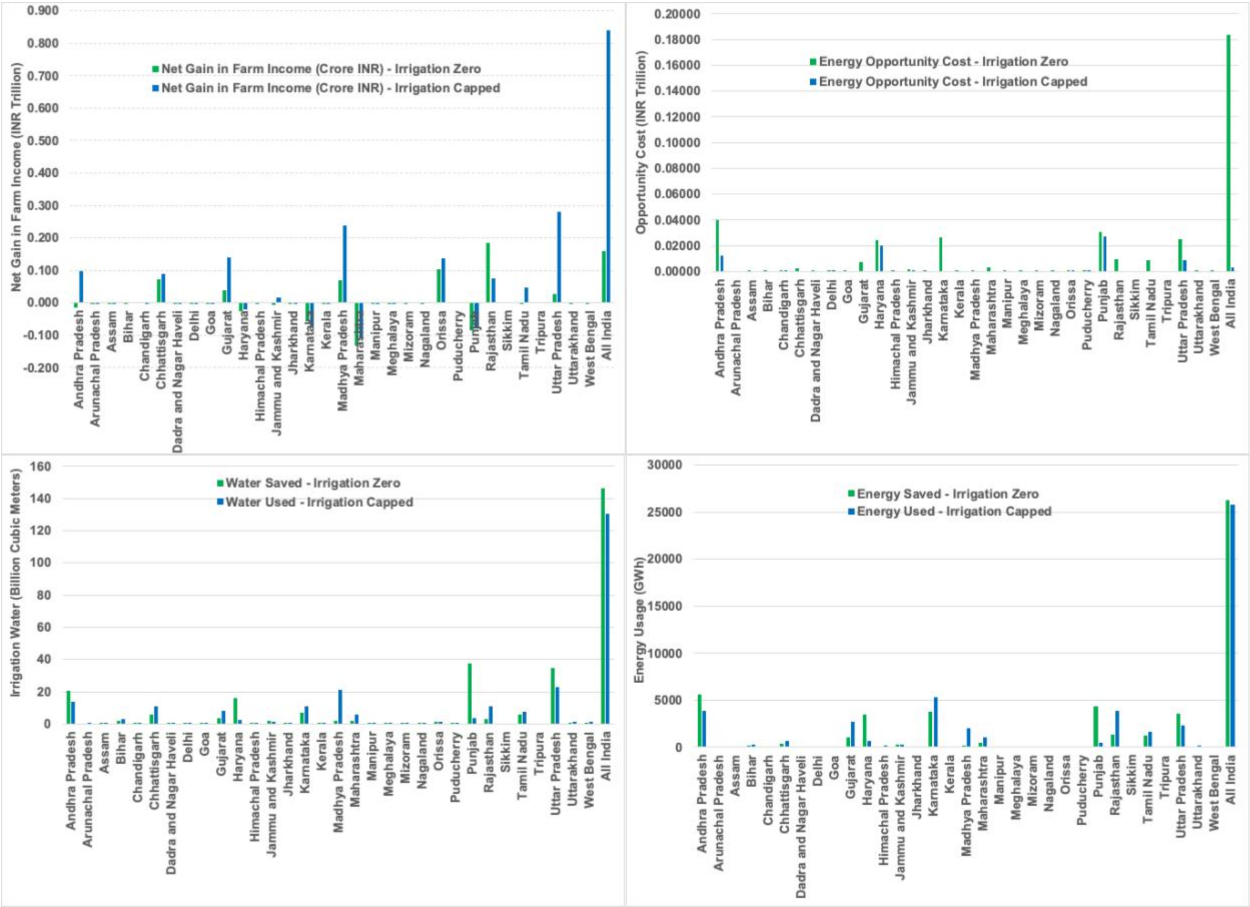

Fig. S4 State-level distribution of net gain in farm income (top left panel), energy opportunity cost (top right panel), water saved/used (bottom left panel), and energy saved/used (bottom right panel) under the “Irrigation Zero” and “Irrigation Capped” scenarios.

Supplementary Note 5

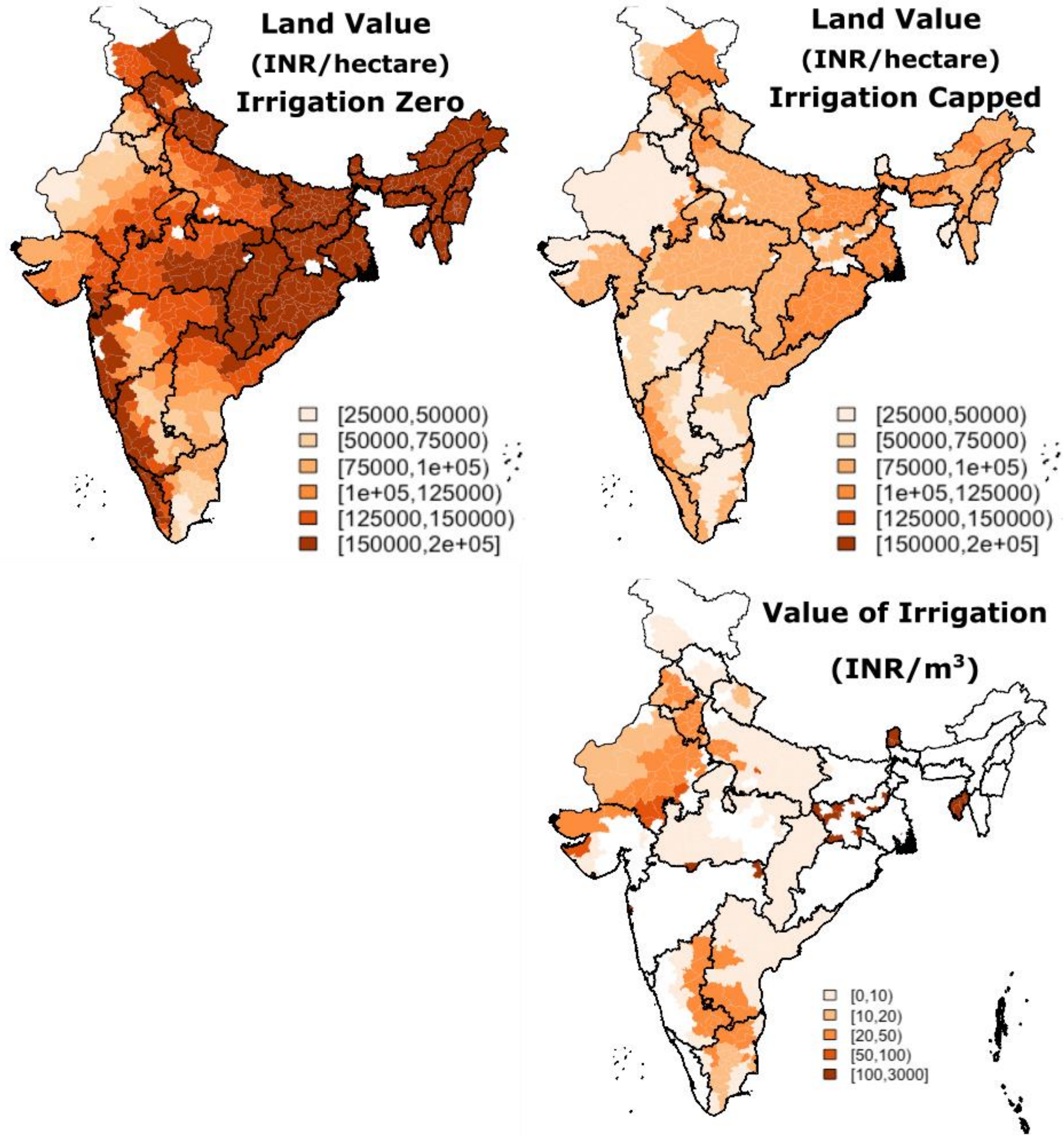

Fig. S5 Shadow prices for the area constraint, i.e., the value of a hectare of arable land under the two scenarios (top panel), and the irrigation constraint (bottom panel).

The shadow prices associated with the constraint on total cropped area per district provide insight into how much incremental value will be added to the optimal solution if cropped area were increased in that location. Figure S5 shows that the highest shadow prices for the land constraint are associated with the regions with the highest rainfall endowment and the lowest variability. This is not a surprise. The values decrease for the “Irrigation Capped” solution relative to the “Irrigation Zero” solution. Adding more land area under irrigated conditions would need to account for the additional cost of groundwater-based irrigation, leading to a lower shadow price since the gain in production and revenue is offset by these costs.

The shadow price for additional irrigation water is highest in the states with high aridity and rainfall variability. This makes sense, reinforcing the notion that access to more water rather than land is important in these states once irrigation is considered. In terms of the PDS design and the local subsidy schemes, the shadow prices can help decide the additional investment in irrigation or in land redevelopment in each district in the country for the optimal solution. We note that for much of the country, the shadow price is zero, and we find that in these areas, irrigation is typically within a posited sustainable limit of 15% of the average annual rainfall per district. This highlights that at least for economically meeting the PDS procurement targets, increasing irrigation is not indicated in these states.

For the other areas, more irrigation would add from INR 20/m^3 to over INR 100/m^3 illustrating the high value of addressing short-term deficits in rainfall relative to the crops selected. This can guide national efforts at developing robust water storage and irrigation storage with appropriate crop and irrigation allocation.

Supplementary Note 6

Table S1 Shadow prices for crop production constraints.

	Shadow Price (INR/Kg)	
**Crop**	**Irrigation Zero**	**Irrigation Capped**
Rice	13.99	11.63
Jowar	11.27	4.12
Bajra	28.02	10.41
Maize	0.74	3.25
Ragi	23.04	2.77
Tur	29.44	0.00
Other Pulses	32.56	0.11
Groundnut	0.00	0.00
Sunflower	85.61	27.13
Soybeen	60.25	29.61
Sesamum	0.00	0.00
Nigerseed	5.26	1.56

We note that the shadow prices for meeting the crop procurement constraint are uniformly lower for the “Irrigation Capped” case relative to the “Irrigation Zero” case. These shadow prices indicate how much the net revenue would increase in the optimal solution per unit reduction in the procurement target. The higher irrigated crop yield and its impact on net revenue lead to a smaller shadow price. Still, in both scenarios, the procurement targets for rice and cereals lead to lower net revenue than would be achieved with the other crops.

Supplementary Note 7

Table S2 List of the different varieties of crops, their minimum support prices, current national Kharif season production and their experimental potential yields used in the study. For each crop variety, the average crop water requirement is shown in parenthesis.

**Crop**	**Minimum Support Price (Rs/100 Kg)**	**Current National Kharif Season Production (Million Tons)**	**Potential Yield (Kg/Ha)**
**Rice (1.8m)**	1750	96.30	4800
**Other Cereals (0.4m)**			
Jowar	2450	1.96	3800
Bajra	1950	9.73	2800
Maize	1700	18.92	6000
Ragi	2897	1.39	2800
**Pulses (0.25m)**			
Tur	5675	4.87	2000
Other Pulses	5600	2.18	2000
**Oilseeds (0.3m)**			
Groundnut	4890	6.05	3300
Sesamum	6249	0.75	1100
Soybean	3399	13.16	2000
Nigerseed	5877	0.09	3000
Sunflower	5388	0.11	2700

Supplementary Note 8

Table S3 Cost of cultivation in different states for the varieties of crops used in the study.

**Cost of Production (Rs/100 Kg)** (Average of 2013-2014, 2014-2015, 2015-2016 Crop Years)												
State	Rice	Jowar	Bajra	Maize	Ragi	Tur	Other Pulses	Groundnut	Sesamum	Soybeen	Nigerseed	Sunflower
Andhra Pradesh	694	698		638	613	3702	1535	2465	4568	3007		3290
Arunachal Pradesh												
Assam	570											
Bihar	627			575		607						
Chandigarh	416			685						2352		
Chhattisgarh	675			404			2352			2037	8233	
Dadra and Nagar Haveli												
Delhi												
Goa												
Gujarat	729		670	1144		2257		1925	3302			
Haryana	662		561									
Himachal Pradesh	310			529								
Jammu and Kashmir												
Jharkhand	650			517								
Karnataka	713	1226	781	772	1659	2570		3096				2904
Kerala	1029											
Madhya Pradesh	705	1041		745		1661	2379		2186	2349	3065	
Maharashtra	1318	1165	1416	1122	1151	2581	3440	3592		2718		3993
Manipur												
Meghalaya												
Mizoram												
Nagaland												
Orissa	663			610		1692	1799	2202	1992		2010	
Puducherry	853	882	644	791	1101		2839	2333	3082			
Punjab	416			685								
Rajasthan		722	434	634			1908	1814	2316	2305		
Sikkim												
Tamil Nadu	853	882	644	791	1101		2839	2333	3082			
Tripura												
Uttar Pradesh	672		409	625		1292	2900		2115			
Uttarakhand	477				211							
West Bengal	724								1859			
National Average	702	945	695	705	973	2045	2444	2470	2722	2461	4436	3396

Supplementary Note 9

Table S4 Nutritional content in the selected crops corresponding to various nutritional goals. The daily recommended intake of these nutrients for an average person from developing countries is given in parenthesis.

	Spectrum of Nutritional Goals (per 100g)									
**Crop**	**Energy (2000 Kcal)**	**Proteins (50 g)**	**Fat (80 g)**	**Iron (21 mg)**	**Calcium (1000 mg)**	**Niacin (15 mg)**	**Folate (170 µg)**	**Vitamin A (500 µg)**	**Vitamin C (30 mg)**	**Ribofalvin (1.4 mg)**
Rice	358	7	1	4	3	4	231	0	0	0
Jowar	339	11	3	4	26	3	0	0	0	0
Bajra	378	11	4	3	22	5	85	0	0	0
Maize	353	9	4	3	10	2	0	0	0	0
Ragi	328	7	1	3	22	5	85	0	0	0
Tur	338	28	1	9	51	3	0	4	6	0
Other Pulses	342	26	1	8	100	2	60	8	4	0
Groundnut	567	26	49	5	92	12	110	0	0	0
Sesamum	573	18	50	15	975	5	97	0	0	0
Soybeen	446	36	20	16	277	2	375	1	6	1
Nigerseed	534	18	42	6	255	3	87	0	1	0
Sunflower	582	19	50	4	70	7	237	0	1	0

Supplementary Note 10

Table S5 State-wide percentage coverage of irrigated area for the selected crops.

State-wise Percent Coverage of Irrigated Area under Principal Crops during 2007-08												
State	Rice	Jowar	Bajra	Maize	Ragi	Tur	Other Pulses	Groundnut	Sesamum	Soybeen	Nigerseed	Sunflower
Andhra Pradesh	97%	8%	29%	44%	32%	0%	2%	18%	20%	9%	20%	41%
Arunachal Pradesh	43%	0%	0%	0%	0%	0%	0%	0%	0%	0%	0%	0%
Assam	4%	0%	0%	0%	0%	0%	0%	0%	0%	0%	0%	0%
Bihar	57%	0%	5%	60%	57%	4%	12%	9%	38%	0%	38%	83%
Chandigarh	99%	0%	94%	64%	70%	93%	88%	21%	87%	0%	87%	100%
Chhattisgarh	33%	5%	2%	5%	2%	0%	7%	15%	5%	2%	5%	95%
Dadra and Nagar Haveli	26%	9%	6%	15%	9%	2%	9%	22%	5%	0%	5%	26%
Delhi	100%	72%	40%	14%	47%	87%	47%	88%	78%	0%	78%	100%
Goa	34%	0%	0%	0%	0%	0%	0%	0%	0%	0%	0%	0%
Gujarat	57%	12%	16%	9%	13%	8%	12%	6%	25%	8%	25%	0%
Haryana	100%	72%	40%	14%	47%	87%	47%	88%	78%	0%	78%	100%
Himachal Pradesh	62%	0%	24%	8%	9%	0%	14%	7%	21%	29%	21%	0%
Jammu and Kashmir	87%	0%	2%	7%	10%	0%	10%	0%	70%	0%	70%	0%
Jharkhand	6%	0%	0%	2%	2%	0%	3%	0%	4%	0%	4%	3%
Karnataka	74%	11%	12%	41%	18%	4%	6%	23%	25%	11%	25%	21%
Kerala	67%	0%	0%	0%	0%	0%	0%	0%	0%	0%	0%	0%
Madhya Pradesh	15%	0%	0%	2%	2%	1%	30%	6%	5%	0%	5%	59%
Maharashtra	26%	9%	6%	15%	9%	2%	9%	22%	5%	0%	5%	26%
Manipur	31%	0%	0%	0%	0%	0%	0%	0%	0%	0%	0%	0%
Meghalaya	31%	0%	0%	0%	0%	0%	0%	0%	0%	0%	0%	0%
Mizoram	18%	0%	0%	0%	0%	0%	0%	0%	0%	0%	0%	0%
Nagaland	45%	0%	0%	0%	0%	0%	0%	0%	52%	0%	52%	55%
Orissa	46%	0%	0%	16%	10%	0%	14%	45%	23%	0%	23%	96%
Puducherry	93%	7%	7%	40%	18%	3%	4%	36%	56%	78%	56%	86%
Punjab	99%	0%	94%	64%	70%	93%	88%	21%	87%	0%	87%	100%
Rajasthan	42%	0%	1%	3%	8%	4%	16%	72%	64%	7%	64%	98%
Sikkim	57%	0%	0%	0%	1%	0%	0%	0%	0%	0%	0%	0%
Tamil Nadu	93%	7%	7%	40%	18%	3%	4%	36%	56%	78%	56%	86%
Tripura	41%	0%	0%	0%	0%	0%	20%	0%	2%	0%	2%	0%
Uttar Pradesh	77%	1%	6%	32%	20%	13%	25%	3%	52%	1%	52%	99%
Uttarakhand	65%	0%	0%	2%	1%	0%	8%	7%	22%	1%	22%	0%
West Bengal	48%	80%	85%	17%	29%	8%	14%	72%	74%	0%	74%	83%
All India	57%	9%	10%	24%	14%	4%	16%	20%	27%	1%	27%	32%
